# Role of tobacco exposure in the course of COVID‐19 disease and the impact of the disease on smoking behavior

**DOI:** 10.1111/crj.13452

**Published:** 2021-10-25

**Authors:** Tahsin Gökhan Telatar, Dilek Karadoğan, Mehmet Halit Baykal, Burcu Aykanat Yurtsever

**Affiliations:** ^1^ Department of Public Health, Faculty of Medicine Recep Tayyip Erdoğan University Rize Turkey; ^2^ Department of Chest Diseases, Faculty of Medicine Recep Tayyip Erdoğan University Rize Turkey; ^3^ Department of Public Health Services Provincial Directorate of Health Rize Turkey

**Keywords:** COVID‐19, follow‐up, quit rate, smoking, sustained abstinence

## Abstract

**Background:**

The effect of COVID‐19 on smoking behavior is not fully known. Studies evaluating the link between smoking and COVID‐19 have controversial results. This study aims to evaluate patients' smoking status with COVID‐19 and the effect of COVID‐19 on smoking behavior.

**Methods:**

Data were collected from 150 COVID‐19 patients with a positive polymerase chain reaction test for SARS‐CoV‐2 between 11 March 2020 and 15 May 2020 in Rize, Turkey. Patients were interviewed by phone calls 2 months after their recovery. After 9 months, a follow‐up was performed for those who quit smoking.

**Results:**

Of the participants, 19 (12.7%) were current smokers before the COVID‐19 diagnosis, and 15 (78.9%) of them stated that they quit smoking after their diagnosis. After nine months of follow‐up, 11 of those 15 participants (57.8%) sustained abstinence.

**Conclusion:**

Smoking cessation rates are high in people with COVID‐19. Besides, the frequency of sustaining abstinence in the long term was also high in these individuals. The COVID‐19 pandemic should be viewed as an open opportunity to strengthen and prioritize smoking cessation activities.

## 1 INTRODUCTION

The first COVID‐19 case in Turkey was seen on 11 March 2020, the same day as the World Health Organization's declaration of pandemic.^1^ The devastating effect of the pandemic in both health and social areas has been observed both globally and in Turkey.^2^ The negative relationship between smoking and many respiratory system diseases, including COVID‐19, has been demonstrated by numerous scientific studies.^3^, ^4^ A review of five studies at the early stages of the pandemic showed that patients with severe disease were higher among smokers.^5^ Liu et al. stated that during COVID‐19, severe pneumonia might be more common, especially among smokers with cigarette‐related comorbidities.^6^ In addition to the health effects of COVID‐19 on smokers, an important issue is its impact on smoking behaviors. One of the most effective interventions in the fight against smoking‐related diseases is increasing smoking cessation rates. Understanding the impact of COVID‐19 on patients' smoking behavior will increase the effectiveness of interventions for this purpose. Although some studies evaluate the smoking cessation status of people after COVID‐19 disease, follow‐up studies evaluating the long‐term persistence of smoking cessation are not yet available in the literature.

The objective of the study is to assess the clinical course of COVID‐19 disease in these people and to determine its relationship with smoking behaviors of COVID‐19 patients diagnosed after the first COVID‐19 patient was recognized in Turkey in the province of Rize between 11 March 2020 and 15 May 2020 and discharged with recovery.

## 2 MATERIALS AND METHODS

**FIGURE 1:**
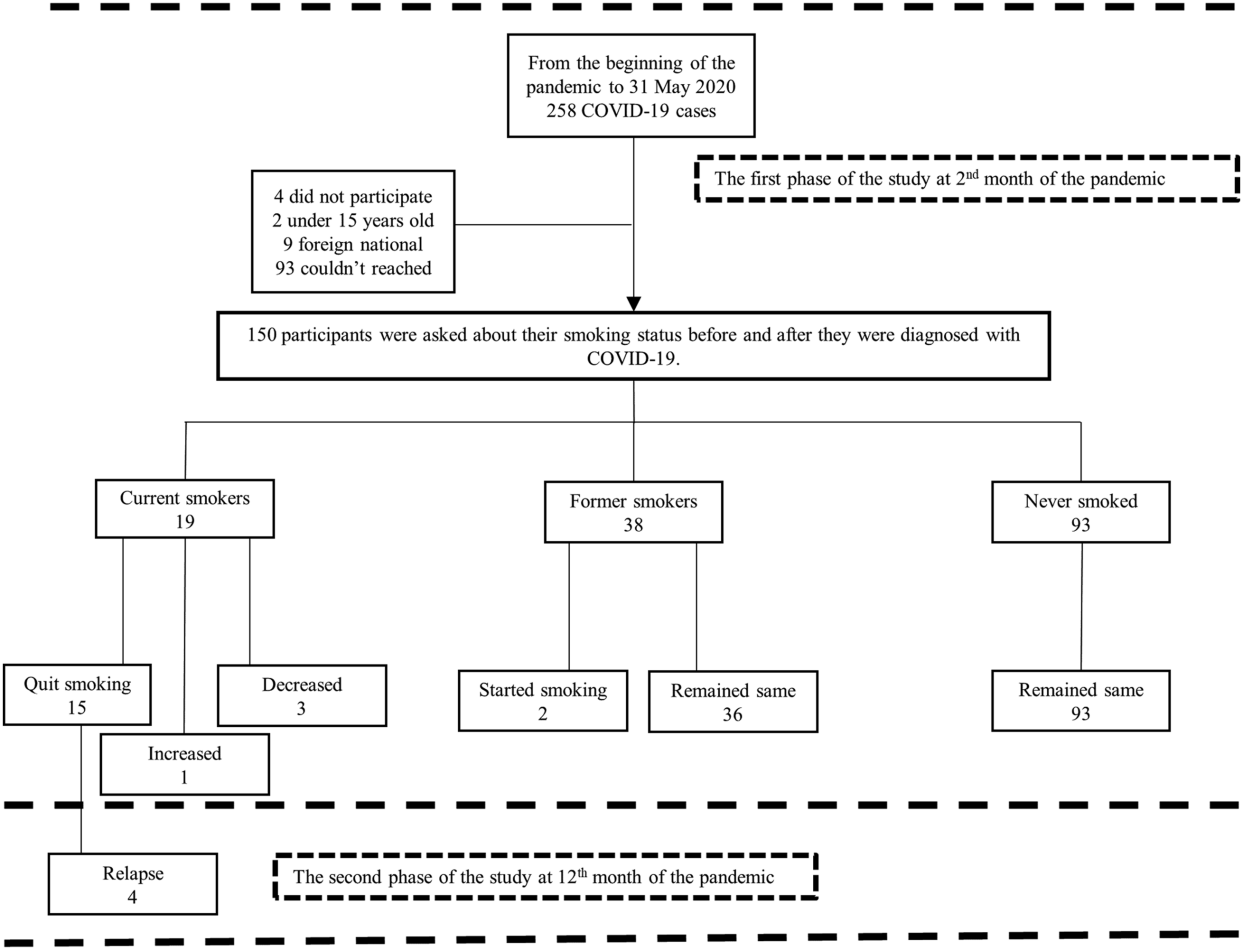
The smoking status of the patients included in the study and the changes observed during the pandemic period

This cross‐sectional study was performed in Rize, a province in the northeast of Turkey. According to the address‐based population registration system data, the population of Rize Province in 2019 was 343 212. A total of 258 COVID‐19 cases were confirmed by polymerase chain reaction test in Rize Province between the date of 11 March 2020, when the first COVID‐19 case was seen in Turkey, and 31 May 2020, when the research started. Those 258 people constitute the universe of the study. No sample was determined for the study, and it was aimed to reach all of the existing patients. Of the patients, four did not want to participate in the study, two were under 15, nine were foreign nationals, and 93 were not included in the study because they could not be reached. The study was conducted with 150 (58.1%) participants. The achieved power for statistical analysis was calculated as 95.7% via GPower 3.1.9.7 software. The data for the study were obtained from the pandemic hospitals, and patients were interviewed by phone calls after 2 months of their recovery. The participants who quit smoking after COVID‐19 diagnoses were interviewed in a follow‐up session at the 12th month of the pandemic (Figure 1).

### 2.1 Statistical analysis

Categorical data were presented as numbers and percentages. Numerical data were presented as means and standard deviations. Relationships between categorical variables were evaluated by the chi‐squared test and Fisher's exact test. Mann–Whitney *U* test was used to evaluate the relationships between smoking status and continuous variables. Some of the analysis results are presented as a supplement. Analysis was conducted by R software Version 4.0.3. Statistical significance level was accepted as *p* < 0.05 in all hypothesis tests.

### 2.2 Ethical issues

The study protocol was approved by the Ethical Board of Recep Tayyip Erdoğan University, Faculty of Medicine University, with the date 4 June 2020 and registration number of 2020/86. All participants were informed about the study, and all volunteered participants' verbal informed consent was obtained.

## 3 RESULTS

The mean age of 150 participants was calculated as 48.1 ± 17.2. Of the participants, 50.0% were women, and the majority (36.7%) were primary school graduates. Nineteen (12.7%) of the participants stated that they were smokers when diagnosed with COVID‐19. Ten (6.7%) of the participants stated that they live alone, and 49 (32.7%) have at least one smoker at home. The frequency of having household members diagnosed with COVID‐19 other than themselves in the participants' homes was found to be 36.7%. Of the participants, 40.7% had at least one chronic comorbid disease (Table 1).

**TABLE 1:** Characteristics of the study population

Variable	Frequency (%) or mean ± standard deviation
Age	48.17 ± 17.23
Gender	
Male	75 (50.0)
Female	75 (50.0)
Education level	
Illiterate	19 (12.7)
Primary schooling	55 (36.7)
Secondary schooling	11 (7.3)
High school	29 (19.3)
University and higher	36 (24.0)
Number of household members	3.65 ± 1.62
Smoker household member	
Present	49 (32.7)
Absent	101 (67.3)
Pre‐COVID‐19 smoking status	
Never smoker	93 (62.0)
Former smoker	38 (25.3)
Current smoker	19 (12.7)
COVID‐diagnosed household member	
Present	55 (36.7)
Absent	95 (63.3)
Comorbid disease	
Present	61 (40.7)
Absent	89 (59.3)

Of the 19 participants who were smokers before being diagnosed with COVID‐19, 15 (78.9%) stated that they quit smoking after getting sick, 2 (10.5%) of them reduced the number of cigarettes they consumed daily, and 1 (5.3%) of them increased the number of cigarettes they consumed daily. Two people who had already quit smoking before being diagnosed with COVID‐19 reported that they started smoking again after they had the disease.

In the follow‐up interview in March 2021, which is the end of the first year of the pandemic and approximately 9 months after the first data collection period, 11 (73.3%) of the 15 participants who stated that they quit smoking after being diagnosed with COVID‐19 continued to be nonsmokers, and four of them (26.7%) started smoking again. The smoking status of the patients and the changes observed during pandemic periods can be seen in Figure 1.

Participants were asked about their thoughts on how smoking affects the course of COVID‐19. Most of the patients (88.0%) think that smoking worsens COVID‐19 progress. Although 15 (11.4%) of those who thought that smoking aggravated the COVID‐19 process were smokers before their illness, 117 (88.6%) were nonsmokers (*p* = 0.174). One participant (0.8%) who thought that smoking aggravated the course of COVID‐19 has increased the number of cigarettes they smoked or started smoking during this period, and 99.2% of them did not change, reduce, or quit their current smoking behavior (*p* = 0.038). Those who believed that smoking negatively affected the course of COVID‐19 had higher sustained abstinence than others (*p* = 0.033) (Table 2).

**TABLE 2:** The relationship between views on the effects of smoking on the COVID‐19 process and smoking behaviors of the participants

Smoking status	Believing that smoking worsens COVID‐19 progress				*p*
	Yes		No		
	n	%	*n*	%	
Before COVID‐19 diagnosis					0.174
Smoker	15	11.4	4	22.2	
Nonsmoker	117	88.6	14	77.3	
Right after COVID‐19 diagnosis					0.038
Not changed, decreased, or quitted	131	99.2	16	88.9	
Increased or started	1	0.8	2	11.1	
At the first year of the pandemic					
Sustained abstinence	10	90.9	1	25.0	0.033
Relapsed	1	9.1	3	75.0	

Information including some sociodemographic characteristics and the clinical status of the participants at the time they were diagnosed with COVID‐19 are presented as Tables S1 and S2.

## 4 DISCUSSION

In this study, the smoking behaviors of people diagnosed with COVID‐19 in the first 3 months of the COVID‐19 pandemic in a city in Turkey were evaluated. Also, the effects of their illness on their smoking behaviors were examined. Of the participants who smoked before being diagnosed with COVID‐19, 78.9% quit smoking after becoming ill. The follow‐up evaluation, held at the end of the first year of the pandemic, revealed that 73.3% of those who quit smoking continued abstinence.

Considering that the prevalence of smoking in the Turkish population is approximately 31.5%,^7^ the prevalence of smoking before COVID‐19 in the study group (12.7%) is low. These findings are consistent with the literature investigating the relationship between COVID‐19 and tobacco exposure during the pandemic. In large‐scale studies examining hospitalized patients with a diagnosis of COVID‐19, the rate of active smoking was significantly lower than the general population.^8^ The low rate of smoking in our study group might have been due to the age distribution of the participants. The majority of smokers in Turkey are in the 15–44 age group.^9^ The average age of our study population is close to 50. Besides, the ratio of women to men was 50% among the participants, and smoking was much less in women compared with men in Turkey.^7^

After getting diagnosed with COVID‐19, 57.8% of the patients quit smoking among the research group, significantly higher than the 7.5% rate in the general population in the United States in 2018.^10^ It is known that people with chronic diseases related to the respiratory system are more prone to quit smoking.^11^ According to the “health belief model,” people are more likely to take a certain health measure if they believe they are at risk of a severe ailment and that the benefits of the action outweigh the costs.^12^ This model may explain the high frequency of smoking cessation after COVID‐19 disease observed in the research group. Similarly, a study conducted in South Asian countries with the participation of approximately 30 000 people showed that there was a 41% reduction in smoking after COVID‐19.^13^

However, there are quite limited studies on changes in smoking behavior attributed to COVID‐19 disease, and it was stated in a recent article that more studies on this subject are needed.^14^ The number of people who want to quit smoking is increasing, and most of them try quitting with self‐efforts without having any external support.^15^ The increase in the frequency of smoking cessation during the pandemic reveals that this challenging process can be turned into an opportunity for smoking cessation activities.^16^ During the COVID‐19 pandemic, many people realized the importance of health‐promoting behaviors and were open to relevant activities, including smoking cessation initiatives.^17^ Unfortunately, like many routine health services, access to smoking cessation treatments remained interrupted, and smokers tried different methods during the pandemic.^14^, ^17^ Even if smoking cessation services continue to serve, people have chosen to stay away from these services in many places due to the pandemic.^18^ In line with the findings of our study, we predict that more successful results can be obtained by focusing on smoking cessation services during the pandemic process.

One of the most important findings of this research is the follow‐up evaluation made in the first year of the pandemic. According to this follow‐up evaluation, which was made with an interval of 9 months, it was determined that of the 15 participants who stated that they quit smoking after getting sick, 11 (73.3%) have continued abstinence. This is a much more successful level than many smoking cessation interventions, including smoking bans, increasing prices, pharmacological approaches, behavioral therapies, and nicotine replacements.^19^ After 9 months, the high level of abstinence strengthens the approach for seeing the pandemic as an opportunity to implement and prioritize the smoking cessation initiatives.

Many studies suggest that smoking is a significant and avoidable risk factor related to the poor prognosis of COVID‐19.^3^ A multinational cross‐sectional study revealed that people who believe that COVID‐19 has more severe progress for smokers have a positive behavioral smoking change during the pandemic.^20^ Our study also presents that people who believe that smoking worsens COVID‐19 progress tend to be nonsmokers or have positive behavioral changes about smoking after being diagnosed with COVID‐19.

Because the study was conducted at the beginning of the pandemic, the small number of participants is the main limitation of our study. But the follow‐up phase makes the findings about abstinence valuable.

Smoking cessation interventions are essential components of tobacco control measures. During this period in which smoking cessation activities are disrupted, turning the pandemic itself into an opportunity by emphasizing its feature that increases the frequency of quitting and revising these services according to the pandemic conditions will increase the success rate of health services concerning smoking cessation.

## ETHICS STATEMENT

The study protocol was approved by the Ethical Board of Recep Tayyip Erdoğan University, Faculty of Medicine University, with the date 4 June 2020 and registration number of 2020/86. All participants were informed about the study, and all volunteered participants' verbal informed consent was obtained.

## CONFLICT OF INTERESTS

None.

## AUTHOR CONTRIBUTIONS

Tahsin Gökhan Telatar has contributed to the conception and the design of the study, collection, analysis, and interpretation of the data, drafting and revising of the manuscript. Dilek Karadoğan has contributed to the conception and the design of the study, collection, analysis, and interpretation of the data, drafting and revising of the manuscript. Mehmet Halit Baykal has contributed to the conception and the design of the study, collection, analysis, and interpretation of the data, drafting and revising of the manuscript. Burcu Aykanat Yurtsever has contributed to the conception and the design of the study, collection, analysis, and interpretation of the data, drafting and revising of the manuscript. All authors approved the final version of the manuscript.

## Supporting information


**Table S1.** COVID‐19 related clinical symptoms of the study populations.

**Table S2.** Relationship of various factors with smoking status before COVID‐19 diagnosis.

## Data Availability

The data that support the findings of this study are openly available in Open Science Framework (OSF) at https://osf.io/n4dwh/?view_only=110843c5abef40ff9cc3ff0236e339b9.
