# Supplementary material for: Role of tobacco exposure in the course of COVID‐19 disease and the impact of the disease on smoking behavior
Source: Clin Respir J. 2021 Oct 25;16(1):57–62. doi: 10.1111/crj.13452 (PMC8652890; doi:10.1111/crj.13452)
Supplement: Supplementary file 1 — Table S1. COVID‐19 related clinical symptoms of the study populations. [file CRJ-16-57-s002.docx]

**Supplement 1.** COVID-19 related clinical symptoms of the study populations.

|  | **Frequency or mean** | **Percent or standard deviation** |
| --- | --- | --- |
| **Fever** |  |  |
| Absent | 96 | 64.0 |
| Present | 54 | 36.0 |
| **Cough** |  |  |
| Absent | 103 | 68.7 |
| Present | 47 | 31.3 |
| **Dyspnea** |  |  |
| Absent | 134 | 89.3 |
| Present | 16 | 10.7 |
| **Headache** |  |  |
| Absent | 119 | 79.3 |
| Present | 31 | 20.7 |
| **Weakness** |  |  |
| Absent | 117 | 78.0 |
| Present | 33 | 22.0 |
| **Body pain** |  |  |
| Absent | 109 | 72.7 |
| Present | 41 | 27.3 |
| **Other symptoms** |  |  |
| Absent | 100 | 66.7 |
| Present | 50 | 33.3 |
| **Number of days with symptoms before treatment** | 4.44 | 5.90 |
| **Number of days with symptoms after treatment** | 8.29 | 12.53 |
| **Treatment** |  |  |
| Monitoring at home | 11 | 7.3 |
| Outpatient care | 3 | 2.0 |
| Inpatient care | 127 | 84.7 |
| Intensive care | 7 | 4.7 |
| Intubated | 2 | 1.3 |

Regarding patients’ symptoms, 36% of them had a fever, 31.3% had a cough, 10.7% had dyspnea, rest of the symptoms are presented in Table 2. The average number of days with symptoms before treatment was 4.44 ± 5.90, and the number of days with symptoms after treatment was 8.29 ± 12.5. Most of the participants were treated as inpatients in the hospital ward (84.7%), 7.3% were followed at home, 4.7% in intensive care, 2% in outpatient treatment, 1.3% were intubated.
