# Supplementary material for: Role of tobacco exposure in the course of COVID‐19 disease and the impact of the disease on smoking behavior
Source: Clin Respir J. 2021 Oct 25;16(1):57–62. doi: 10.1111/crj.13452 (PMC8652890; doi:10.1111/crj.13452)
Supplement: Supplementary file 2 — Table S2. Relationship of various factors with smoking status before COVID‐19 diagnosis. [file CRJ-16-57-s001.docx]

**Supplement 2.** Relationship of various factors with smoking status before COVID-19 diagnosis.

|  | **Non-smoker (past and never smokers)** | | **Current smoker** | | **p** |
| --- | --- | --- | --- | --- | --- |
|  | **N or mean** | **% or SD** | **N or mean** | **% or SD** |  |
| **Age** | 46.05 | 14.15 | 46.05 | 14.15 | 0.569 |
| **Gender** |  |  |  |  | 0.027 |
| Male | 61 | 46.6 | 14 | 73.7 |  |
| Female | 70 | 53.4 | 5 | 26.3 |  |
| **Education level** |  |  |  |  | - |
| Illiterate | 19 | 14.5 | 0 | 0.0 |  |
| Primary school | 47 | 35.9 | 8 | 42.1 |  |
| Secondary school | 8 | 6.1 | 3 | 15.8 |  |
| High school | 25 | 19.1 | 4 | 21.1 |  |
| University and over | 32 | 24.4 | 4 | 21.1 |  |
| **Pack/year** | 18.79 | 20.05 | 22.29 | 13.74 | 0.503 |
| **Number of household members** | 3.64 | 1.62 | 3.68 | 1.70 | 0.914 |
| **Other smokers in the household** |  |  |  |  | 0.144 |
| Present | 40 | 30.5 | 9 | 47.4 |  |
| Absent | 91 | 69.5 | 10 | 52.6 |  |
| **Smoking status after COVID** |  |  |  |  | - |
| No difference | 129 | 98.5 | 0 | 0.0 |  |
| Decreased | 0 | 0.0 | 3 | 15.8 |  |
| Quitted | 0 | 0.0 | 15 | 78.9 |  |
| Increased | 2 | 1.5 | 1 | 5.3 |  |
| **Is smoking a disadvantage for COVID?** |  |  |  |  | 0.249 |
| Yes | 117 | 89.3 | 15 | 78.9 |  |
| No | 14 | 10.7 | 4 | 21.1 |  |
| **Number of days with symptoms before treatment** | 4.64 | 6.18 | 2.77 | 1.92 | 0.281 |
| **Number of days with symptoms after treatment** | 8.26 | 12.06 | 8.54 | 16.50 | 0.940 |
| **Treatment** |  |  |  |  | - |
| Monitoring at home | 10 | 7.6 | 1 | 5.3 |  |
| Outpatient care | 3 | 2.3 | 0 | 0.0 |  |
| Inpatient care | 111 | 84.7 | 16 | 84.2 |  |
| Intensive care | 7 | 5.3 | 0 | 0.0 |  |
| Intubated | 0 | 0.0 | 2 | 10.5 |  |
| **Treatment duration (day)** | 12.15 | 7.36 | 14.05 | 20.09 | 0.432 |
| **Intubation** |  |  |  |  | 0.015 |
| Absent | 131 | 100.0 | 17 | 89.5 |  |
| Present | 0 | 0.0 | 2 | 10.5 |  |
| **Other households with COVID diagnosis** |  |  |  |  | 0.316 |
| Present | 50 | 38.2 | 5 | 26.3 |  |
| Absent | 81 | 61.8 | 14 | 73.7 |  |
| **Chronic disease** |  |  |  |  | 0.891 |
| Present | 53 | 40.5 | 8 | 42.1 |  |
| Absent | 78 | 59.5 | 11 | 57.9 |  |

Those with a "-" sign where the p-value is in place are variables that the chi-square test could not be conducted because of inadequate data.

When the findings of active smokers and other patients were compared, 73% of active smokers were male, and 46.6% of non-smokers were male (p =0.027). While 10.5% of the current smoker group was intubated, there were no intubated patients among non-smokers (p= 0.015).
